# Circulating Tumor DNA (ctDNA) Clearance May Predict Treatment Response in Neoadjuvant Colorectal Cancer Management

**DOI:** 10.3390/jcm13061684

**Published:** 2024-03-14

**Authors:** Britney Niemann, John Moise, Michael Sestito, Midhun Malla, Kevin Train, Douglas Murken, Keri Mayers, Emily Groves, Mary Garland-Kledzik

**Affiliations:** 1 Department of Surgery, West Virginia University, Morgantown, WV 26505, USA; brharris1@hsc.wvu.edu (B.N.); michael.sestito@hsc.wvu.edu (M.S.); kevin.train@hsc.wvu.edu (K.T.); douglas.murken@hsc.wvu.edu (D.M.); keri.mayers@hsc.wvu.edu (K.M.); emily.groves@hsc.wvu.edu (E.G.); 2 School of Medicine, West Virginia University, Morgantown, WV 26505, USA; 3 Division of Hematology and Oncology, University of Alabama, 2000 6th Avenue South, Floor 5, Birmingham, AL 35233, USA

**Keywords:** circulating tumor DNA (ctDNA), neoadjuvant, colorectal cancer

## Abstract

**Background:** Circulating tumor DNA (ctDNA) is extracellular DNA released by tumors and has been proposed as a marker of residual disease as well as a predictor of disease recurrence in the adjuvant setting. However, data are lacking on the utility of this biomarker in the neoadjuvant setting. **Methods:** We performed a retrospective study of stage III and IV colorectal cancer patients receiving neoadjuvant treatment at a single institution. **Results:** Seventeen patients converted from a positive pre-neoadjuvant ctDNA to a negative ctDNA prior to surgery. Five patients remained persistently positive despite systemic treatment. ctDNA conversion was found to be associated with a higher incidence of favorable treatment effect scores on final surgical pathology. There was no difference in recurrence-free survival in this small population. Furthermore, no added benefit was identified for patients receiving additional neoadjuvant therapy after the time of positive to negative ctDNA conversion. **Conclusions:** This study highlights the potential utility of ctDNA and the need for prospective trials in the neoadjuvant setting to monitor treatment response and guide decisions on treatment duration.

## 1. Introduction

Circulating tumor DNA (ctDNA) is fragmented, extracellular DNA that is released by tumor cells and represents a promising predictive and prognostic biomarker in the clinical management of solid tumors. These DNA fragments contain patient-specific genetic alterations, which allows for differentiation from other circulating cell-free DNA. With a half-life ranging from 30 min to 2.5 h, ctDNA reflects in real-time the extent of tumor burden, allowing for more personalized and targeted cancer care [1,2]. While ctDNA can vary significantly based on the individual and type of cancer, multiple studies have shown a correlation between ctDNA level and tumor size, histological grade, and the presence of microscopic residual disease [1,2,3,4,5,6]. One prospective clinical trial demonstrated that patients with positive postoperative ctDNA were seven times more likely to relapse. Similarly, patients with positive ctDNA after adjuvant therapy were 17 times more likely to relapse [7]. The GALAXY trial also demonstrated that ctDNA trends in relation to adjuvant therapy were predictive of recurrence-free survival (RFS), highlighting ctDNA as a potential marker of treatment response [8].

Colorectal cancer (CRC) is the second leading cause of cancer-related deaths in the United States, with over half of patients presenting with stage III or IV disease [9,10]. Surgery and systemic therapy have been the mainstay of treatment, with recent evidence showing the benefit of systemic therapy delivered in the neoadjuvant setting. For instance, the RAPIDO and PRODIGE 23 trials found higher pathologic complete response (pCR) rates and lower disease-related treatment failure in rectal cancer patients after neoadjuvant therapy [11,12,13,14]. PRODIGE 23, in particular, found improved RFS, metastasis-free survival, and overall survival (OS) after 7 years. Similarly, the FOxTROT trial demonstrated decreased 2-year recurrence with neoadjuvant therapy in patients with nonmetastatic colon cancer [15]. In the setting of metastatic disease requiring cytoreductive surgery (CRS) and hyperthermic intraperitoneal chemotherapy (HIPEC), neoadjuvant therapy was associated with higher complete cytoreduction and survival [16,17].

Although the benefits of neoadjuvant treatment have been shown, the best treatment regimen and duration are unclear, as demonstrated by the treatment variability in neoadjuvant clinical trials. Given the success of ctDNA in the adjuvant setting as an indicator of treatment response and recurrence, this study examines the utility of ctDNA during neoadjuvant management of CRC.

## 2. Materials and Methods

### 2.1. Patient Selection

After approval was obtained from the institutional review board, a retrospective review of patients aged 18 or older at a single institution with colorectal cancer from 2020 to 2022 was performed. Patients with stage III or IV disease, as defined by NCCN guidelines, as well as at least one ctDNA testing time point were included in the study [18]. We included patients presenting with a recurrence from a previously treated CRC. Patient demographics, including age, sex, and BMI, as well as treatment regimens, both in the neoadjuvant and adjuvant settings, were collected.

### 2.2. ctDNA Measurement

Patient tumor samples were obtained via biopsy for whole exome sequencing of multiple patient-specific somatic single-nucleotide variants. Two or more variants were required to ensure adequate specificity. Patient plasma samples were collected and analyzed by Natera Inc (Austin, TX, USA). [19]. Tumor-informed ctDNA was recorded as mean tumor molecules per millimeter of plasma (MTM/mL). In this study, real-world data from commercial ctDNA testing was retrospectively analyzed.

### 2.3. Outcomes

Patients were categorized based on the availability of ctDNA at different treatment time points. The primary analysis included patients with a minimum of one ctDNA prior to initiation of neoadjuvant therapy and an additional ctDNA preoperatively. Patients included in the secondary analysis had a minimum of a postoperative ctDNA level and/or ctDNA at the time of recurrence. To further understand the utility of ctDNA in the neoadjuvant setting, tumor characteristics on final surgical pathology were examined, including lymphovascular invasion, perineural invasion, the pCR rate, and final resection margin status. The treatment effect score, as defined by the Modified Ryan scheme for tumor regression, was also reported where patients received a score of 0 (no viable cancer cells; complete response), 1 (single cells or rare small groups of cells; near complete response), 2 (residual cancer with evident tumor regression but more than single cells or rare small groups of cancer cells; partial response), or 3 (extensive residual cancer with no evident tumor regression; poor or no response) [20]. Lastly, RFS and OS were calculated for all patients. RFS was defined as the time from surgery to the day of recurrence diagnosis. Recurrence was determined by diagnostic imaging. Patients who had no recurrence at time of last follow-up were censored. These outcomes were also all examined in relation to ctDNA in the adjuvant setting.

### 2.4. Statistical Analyses

Prism version 10.1.1 was utilized for all statistical analyses. Parametric data were described using the mean/standard deviation and analyzed using an unpaired *t* test. Nonparametric data were described using the median/interquartile range and analyzed with a Mann–Whitney test. Categorical data were organized in contingency tables and analyzed using Fisher’s exact test given the small sample numbers. Kaplan–Meyer curves were generated for RFS and OS.

## 3. Results

### 3.1. ctDNA Response to Systemic Neoadjuvant Treatment

**Figure 1:**
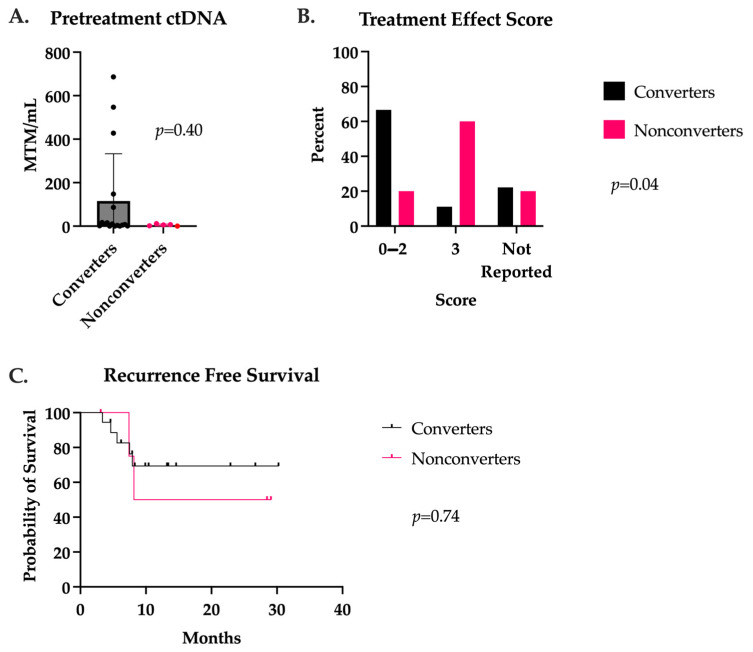
(**A**) ctDNA levels prior to neoadjuvant treatment initiation in patients with positive to negative ctDNA conversion (converters) and patients with persistently positive ctDNA in the neoadjuvant setting (nonconverters). (**B**) Treatment effect score as defined by the Modified Ryan scheme for tumor regression, 0—2 correlated to a complete to partial treatment response and 3 to a poor or absent response. (**C**) Recurrence-free survival after a median follow-up of 14.5 months in converters and nonconverters.

Fifty-two stage III or IV CRC patients with available ctDNA data were identified for this study. Twenty-four patients had ctDNA available preoperatively, whereas 36 patients had postoperative ctDNA available. Between the time of neoadjuvant treatment initiation and surgical intervention, 17 patients had a conversion of their ctDNA levels from positive to negative (termed converters), while seven patients remained positive at the time of surgery (termed nonconverters). There were no significant differences in baseline patient characteristics or treatment regimens between the groups, as demonstrated in Table 1. Interestingly, there was a notable trend in the proportion of female patients who were converters, 10 of 11 (90.9%), which was seen to a lesser degree in male patients, 7 of 11 (63.6%) included in primary analysis. The location of metastases in the converters group included the liver (n = 3) and lung (n = 1). The liver was the only site of visceral metastases in the nonconverters group. The converters had a median baseline ctDNA of 9.8 versus 5.8 MTM/mL in the nonconverters (*p* = 0.40), with more variability seen in the converter group (Figure 1A). All the converters proceeded to have an R0 resection. However, two of the nonconverters were found to experience a progression of disease at the time of surgery and were unable to undergo resection. There were no differences in final pathology between groups for lymphovascular invasion, perineural invasion, or pCR (Table 2). Converters were found to have more favorable treatment effect scores (0–2), correlating with complete to partial responses (*p* = 0.04) (Figure 1B). After a median follow-up of 14.5 months, there were no deaths in either group, and no difference in RFS (*p* = 0.74) was observed (Figure 1C).

### 3.2. Duration of Neoadjuvant Therapy in Converters

**Figure 2:**
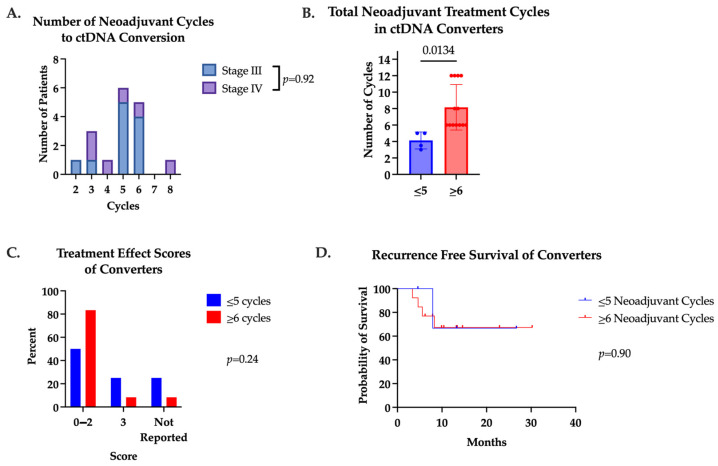
(**A**) Number of cycles patients received prior to positive to negative ctDNA conversion. Median number of cycles was 5. Converters were divided into two groups based on total number of neoadjuvant cycles received (≤5 cycles or ≥6 cycles). (**B**) Total number of neoadjuvant cycles converters received in converters. (**C**) Treatment effect scores of converters receiving ≤5 cycles or ≥6 cycles. (**D**) Recurrence-free survival of converters who received ≤5 cycles versus ≥6 cycles neoadjuvant cycles.

Patients who underwent a positive to negative ctDNA conversion (converters) received a median of five (range two to eight) cycles of systemic therapy prior to ctDNA conversion (Figure 2A). For this reason, we divided converters into those receiving five or fewer cycles of systemic therapy (n = 4) and those receiving six or more cycles (n = 13) to evaluate the impact of systemic therapy duration. There was no difference in pretreatment ctDNA between these two groups. As expected, patients in the ≥6 cycles group received significantly more cycles of therapy with a max of 12 cycles (Figure 2B). One (25%) patient in the ≤5 cycles group had a pCR, as well as one (7.7%) patient in the ≥6 cycles group. Treatment effect scores and RFS were also similar between groups (Figure 2C,D).

**Figure 3:**
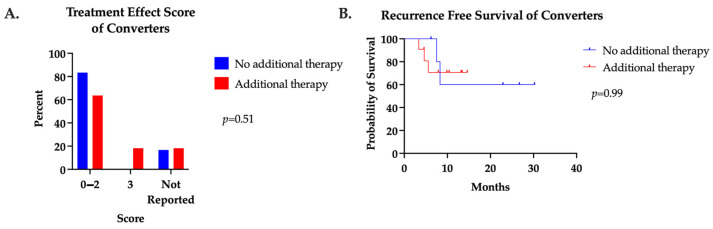
(**A**) Treatment effect score and (**B**) recurrence-free survival of converters who received additional neoadjuvant systemic therapy after positive to negative ctDNA conversion compared to those who did not.

We also examined the impact of additional neoadjuvant systemic therapy after patients underwent a positive to negative ctDNA conversion. Eleven (65%) converters received additional neoadjuvant cycles after ctDNA conversion. A median of four additional cycles (range one to nine) were administered in this group. No difference in pCR, treatment effect score, or RFS were found between groups (Figure 3).

### 3.3. ctDNA and Recurrence

**Figure 4:**
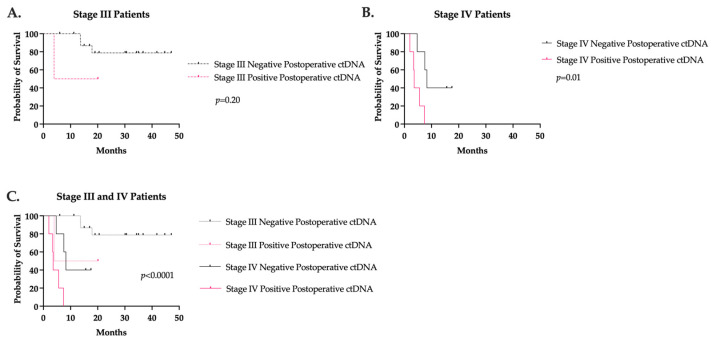
Recurrence-free survival based on postoperative ctDNA value in (**A**) stage III and (**B**) stage IV colorectal cancer. (**C**) Stage III and IV recurrence-free survival curves together.

Postoperative ctDNA was available for 32 CRC patients. Nineteen (59%) patients were stage III with negative ctDNA, two (6%) stage III with positive ctDNA, five (16%) stage IV with negative ctDNA, and six (19%) stage IV with positive ctDNA. All patients underwent an R0 resection. While no difference in RFS was seen in stage III patients, positive postoperative ctDNA was associated with a significantly lower RFS in stage IV patients compared to those with negative postoperative ctDNA (3.6 months versus 8.3 months; *p* = 0.01) (Figure 4). Interestingly, one stage III colon cancer patient had a persistently positive ctDNA for 21.5 months without evidence of recurrence.

**Figure 5:**
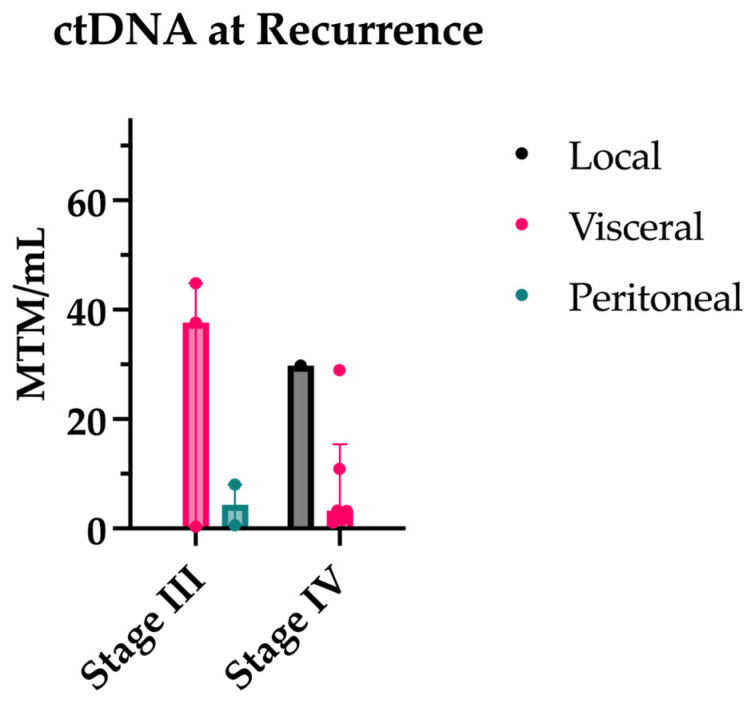
ctDNA levels at time of recurrence separated by original stage and site of recurrence.

Twelve patients (five stage III, seven stage IV) had a ctDNA at the time of recurrence, with all but one stage III and one stage IV patient having ctDNA levels prior to recurrence for comparison. All patients had a positive ctDNA level at the time of recurrence. All patients with prior ctDNAs for comparison experienced an increase in ctDNA at or near the time of recurrence. In fact, 90% of patients had an increase in ctDNA prior to the radiologic evidence of disease. The median lead time was 3.13 months. ctDNA levels at the time of recurrence are shown by the recurrence site in Figure 5.

Lastly, five patients had negative ctDNAs following neoadjuvant therapy and decided to pursue a watch-and-wait approach. Serial ctDNAs were obtained in addition to imaging for surveillance. Recurrence occurred in two patients, one with a local recurrence after 26.7 months and the other with a distant recurrence after 9.1 months. At the time of the recurrence, both patients developed a positive ctDNA.

## 4. Discussion

Emerging data support the prognostic benefit of neoadjuvant therapy in multiple cancers, including CRC [11]. However, the ideal regimen and duration of therapy have yet to be determined. Through tumor sequencing, we know even tumors of the same origin are extremely complex and variable, altering an individual’s response to particular systemic therapies [21,22,23]. Given this, it is likely patients would benefit from a more personalized approach to their neoadjuvant care. To accomplish this, we are in need of tools to evaluate the response to treatment. In the adjuvant setting, ctDNA was shown to detect microscopic residual disease and predict recurrence, which we found to be replicated here in our patient population with stage IV disease. Furthermore, the Galaxy trial found that ctDNA conversion from positive to negative in the setting of adjuvant therapy was associated with a prolonged DFS, indicating that ctDNA may be useful in monitoring treatment response [8].

Despite the emerging evidence behind ctDNA in the adjuvant setting, there remains a paucity of data in the neoadjuvant setting. Therefore, we retrospectively evaluated tumor-informed ctDNA trends in colorectal patients receiving neoadjuvant therapy. We found patients who converted from positive to negative ctDNA had significantly higher proportions of favorable treatment effect scores on pathologic examination, indicating that ctDNA clearance may predict tumor response to neoadjuvant therapy. This could be instrumental in the early detection of poor treatment response in the neoadjuvant setting, allowing for alternative regimens to be explored. Additionally, when examining the delivery of additional systemic therapy after ctDNA conversion, we found no additional benefit. As patients can become more deconditioned during chemotherapy, this highlights the possible role of ctDNA in also guiding the duration of neoadjuvant treatment and determining when patients are appropriate for surgery. Limiting unnecessary toxicity and optimizing the nutritional and functional status of patients is critical prior to surgery. ctDNA may, therefore, help reduce the risk of overtreatment while simultaneously identifying those with aggressive tumor biology who would not benefit from surgical resection and its associated morbidity. While there was no difference in RFS, this study was underpowered to detect such a difference.

One concern with neoadjuvant treatment in resectable stage IV disease is the potential for disease progression prior to surgical intervention. As previously discussed, ctDNA levels can vary based on the individual and tumor site, including the sites of metastases. In particular, there have been variable reports of the sensitivity of ctDNA in the setting of peritoneal metastases [24,25,26,27,28]. Peritoneal metastases are unique in that they are often poorly vascularized and, thus, disconnected from the systemic circulation [29]. About 17% of stage IV CRC patients will have peritoneal metastases, making this an important point to consider when evaluating systemic markers to guide treatment decisions [18]. In our patient cohort, there were only two patients with only peritoneal disease recurrence. Both of these patients had a correlating increase in tumor-informed ctDNA at the time of recurrence. Larger scale studies are needed to investigate this further.

This study is limited by several factors. First is the small sample size. Given the lack of ctDNA protocol at our institution, the data available for ctDNA trends in the neoadjuvant setting were limited. Therefore, many of the outcomes were underpowered to detect a true difference. For this reason, stage III and IV patients were combined in an attempt to increase the sample size. Future studies with larger cohorts should evaluate these groups separately as the tumor biology may differ, impacting ctDNA. Second, we did not collect information on neoadjuvant therapy dose reductions. This is important to consider when interpreting the neoadjuvant data, such as the number of cycles needed for conversion and the benefit of additional cycles after conversion. Lastly, this study was retrospective in nature. Therefore, additional larger-scale and prospective studies are needed to confirm and expand the findings described here.

## 5. Conclusions

In this retrospective hypothesis-generating study, we analyzed the potential benefits of ctDNA use in the neoadjuvant setting. In our analyses of real world ctDNA testing in CRC cancers, ctDNA clearance may predict pathologic treatment response in the neoadjuvant colorectal cancer management as well as provide prognostic information that is important for surgical discussions. As ctDNA has become integral to the adjuvant care of these patients, we look forward to prospective studies powered to detect the optimum duration of therapy prior to curative intent resection.

## Figures and Tables

**Table 1:** Patient demographics.

	Converters N = 17	Nonconverters N = 5	*p* Value
Age, mean years (SD)	54.0 (12.4)	54.6 (12.2)	0.93
Female, n (%)	10.0 (58.8)	1.0 (20.0)	0.31
BMI, mean (SD)	30.6 (6.2)	29.5 (6.1)	0.74
Cancer Diagnosis, n (%)			0.89
Stage III Colon Cancer	4.0 (23.5)	0.0 (0.0)	
Stage III Rectal Cancer	7.0 (41.8)	3.0 (60.0)	
Stage IV Colon Cancer	3.0 (17.6)	1.0 (20.0)	
Stage IV Rectal Cancer	3.0 (17.6)	1.0 (20.0)	
Location of Metastases, n (%)			0.36
Viscera	1.0 (16.7)	1.0 (50.0)	
Peritoneum	2.0 (33.3)	1.0 (50.0)	
Viscera and Peritoneum	3.0 (50.0)	0.0 (0.0)	
Neoadjuvant Radiation, n (%)	9.0 (52.9)	4.0 (80.0)	0.36
Neoadjuvant Systemic Treatment, n (%)			0.68
FOLFIRINOX	6.0 (35.3)	1.0 (20.0)	
FOLFOXIRI	1.0 (5.9)	0.0 (0.0)	
FOLFOX	0.0 (0.0)	1.0 (20.0)	
CAPEOX	3.0 (17.6)	2.0 (40.0)	
Nivolumab + Ipillimumab	2.0 (11.8)	0.0 (0.0)	
Chemotherapy + Target-Directed Therapy	4.0 (23.5)	1.0 (20.0)	
Combination of Chemotherapies	1.0 (5.9)	0.0 (0.0)	
Total Number Neoadjuvant Therapy Cycles, median (IQR)	6.0 (5.5–10.0)	8.5 (6.0–12.3)	0.25
Duration of Neoadjuvant Therapy, median months (IQR)	7.2 (6.6–8.6)	8.7 (6.2–10.3)	0.15
CEA at diagnosis, median ng/mL (IQR)	5.1 (1.9–28.6)	3.2 (1.7–5.4)	0.35

**Table 2:** Outcomes.

	Converters	Nonconverters	*p* Value
R0 resection, n (%)	18.0 (100.0)	5.0 (100.0)	0.99
Treatment effect score, n (%)			0.04
Complete to partial (score 0–2)	12.0 (66.7)	1.0 (20.0)	
Poor or absent (score 3)	2.0 (11.1)	3.0 (60.0)	
Not reported	4.0 (22.2)	1.0 (20.0)	
Lymphovascular invasion present, n (%)	8.0 (47.1)	3.0 (60.0)	0.99
Perineural invasion present, n (%)	5.0 (35.7)	3.0 (60.0)	0.60
Pathologic Complete Response, n (%)	2.0 (11.1)	0.0 (0.0)	0.99

## Data Availability

The data presented in this study are available upon request from the corresponding author.

## References

[1] Yao W., Mei C., Nan X., Hui L. (2016). Evaluation and comparison of in vitro degradation kinetics of DNA in serum, urine and saliva: A qualitative study. Gene.

[2] Diehl F., Schmidt K., Choti M.A., Romans K., Goodman S., Li M., Thornton K., Agrawal N., Sokoll L., Szabo S.A. (2008). Circulating mutant DNA to assess tumor dynamics. Nat. Med..

[3] Thierry A.R., Mouliere F., Gongora C., Ollier J., Robert B., Ychou M., Del Rio M., Molina F. (2010). Origin and quantification of circulating DNA in mice with human colorectal cancer xenografts. Nucleic Acids Res..

[4] Bettegowda C., Sausen M., Leary R.J., Kinde I., Wang Y., Agrawal N., Bartlett B.R., Wang H., Luber B., Alani R.M. (2014). Detection of circulating tumor DNA in early- and late-stage human malignancies. Sci. Transl. Med..

[5] Ohara S., Suda K., Sakai K., Nishino M., Chiba M., Shimoji M., Takemoto T., Fujino T., Koga T., Hamada A. (2020). Prognostic implications of preoperative versus postoperative circulating tumor DNA in surgically resected lung cancer patients: A pilot study. Transl. Lung Cancer Res..

[6] Zhu J.W., Wong F., Szymiczek A., Ene G.E.V., Zhang S., May T., Narod S.A., Kotsopoulos J., Akbari M.R. (2023). Evaluating the Utility of ctDNA in Detecting Residual Cancer and Predicting Recurrence in Patients with Serous Ovarian Cancer. Int. J. Mol. Sci..

[7] Reinert T., Henriksen T.V., Christensen E., Sharma S., Salari R., Sethi H., Knudsen M., Nordentoft I., Wu H.T., Tin A.S. (2019). Analysis of Plasma Cell-Free DNA by Ultradeep Sequencing in Patients With Stages I to III Colorectal Cancer. JAMA Oncol..

[8] Kotani D., Oki E., Nakamura Y., Yukami H., Mishima S., Bando H., Shirasu H., Yamazaki K., Watanabe J., Kotaka M. (2023). Molecular residual disease and efficacy of adjuvant chemotherapy in patients with colorectal cancer. Nat. Med..

[9] National Cancer Institute Cancer Stat Facts: Common Cancer Sites. https://seer.cancer.gov/statfacts/html/common.html.

[10] SEER*Explorer: An Interactive Website for SEER Cancer Statistics. https://seer.cancer.gov/statistics-network/explorer/.

[11] Conroy T., Bosset J.F., Etienne P.L., Rio E., François É., Mesgouez-Nebout N., Vendrely V., Artignan X., Bouché O., Gargot D. (2021). Neoadjuvant chemotherapy with FOLFIRINOX and preoperative chemoradiotherapy for patients with locally advanced rectal cancer (UNICANCER-PRODIGE 23): A multicentre, randomised, open-label, phase 3 trial. Lancet Oncol..

[12] Conroy T., Etienne P.-L., Rio E., Evesque L., Mesogouez-Nebout N., Vendrely V., Artignan X., Bouche O., Boileve A., Delaye M. (2023). Total neoadjuvant therapy with mFOLFIRINOX versus preoperative chemoradiation in patients with locally advanced rectal cancer: 7-year results of PRODIGE 23 phase III trial, a UNICANCER GI trial. J. Clin. Oncol..

[13] Bahadoer R.R., Dijkstra E.A., van Etten B., Marijnen C.A.M., Putter H., Kranenbarg E.M., Roodvoets A.G.H., Nagtegaal I.D., Beets-Tan R.G.H., Blomqvist L.K. (2021). Short-course radiotherapy followed by chemotherapy before total mesorectal excision (TME) versus preoperative chemoradiotherapy, TME, and optional adjuvant chemotherapy in locally advanced rectal cancer (RAPIDO): A randomised, open-label, phase 3 trial. Lancet Oncol..

[14] Dijkstra E.A., Nilsson P.J., Hospers G.A.P., Bahadoer R.R., Meershoek-Klein Kranenbarg E., Roodvoets A.G.H., Putter H., Berglund Å., Cervantes A., Crolla R.M.P.H. (2023). Locoregional Failure During and After Short-course Radiotherapy Followed by Chemotherapy and Surgery Compared With Long-course Chemoradiotherapy and Surgery: A 5-Year Follow-up of the RAPIDO Trial. Ann. Surg..

[15] Morton D., Seymour M., Magill L., Handley K., Glasbey J., Glimelius B., Palmer A., Seligmann J., Laurberg S., Murakami K. (2023). Preoperative Chemotherapy for Operable Colon Cancer: Mature Results of an International Randomized Controlled Trial. J. Clin. Oncol..

[16] Zhou S., Jiang Y., Liang J., Pei W., Zhou Z. (2021). Neoadjuvant chemotherapy followed by hyperthermic intraperitoneal chemotherapy for patients with colorectal peritoneal metastasis: A retrospective study of its safety and efficacy. World J. Surg. Oncol..

[17] Flood M.P., Kong J.C.H., Wilson K., Mohan H., Waters P.S., McCormick J.J., Warrier S.K., Tie J., Ramsay R., Michael M. (2022). The Impact of Neoadjuvant Chemotherapy on the Surgical Management of Colorectal Peritoneal Metastases: A Systematic Review and Meta-Analysis. Ann. Surg. Oncol..

[18] National Comprehensive Cancer Network NCCN Guidelines: Colon Cancer. https://www.nccn.org/professionals/physician_gls/pdf/colon.pdf.

[19] Natera. https://www.natera.com/oncology/signatera-advanced-cancer-detection/.

[20] Ryan R., Gibbons D., Hyland J.M., Treanor D., White A., Mulcahy H.E., O’Donoghue D.P., Moriarty M., Fennelly D., Sheahan K. (2005). Pathological response following long-course neoadjuvant chemoradiotherapy for locally advanced rectal cancer. Histopathology.

[21] Misale S., Yaeger R., Hobor S., Scala E., Janakiraman M., Liska D., Valtorta E., Schiavo R., Buscarino M., Siravegna G. (2012). Emergence of KRAS mutations and acquired resistance to anti-EGFR therapy in colorectal cancer. Nature.

[22] Woolston A., Khan K., Spain G., Barber L.J., Griffiths B., Gonzalez-Exposito R., Hornsteiner L., Punta M., Patil Y., Newey A. (2019). Genomic and Transcriptomic Determinants of Therapy Resistance and Immune Landscape Evolution during Anti-EGFR Treatment in Colorectal Cancer. Cancer Cell.

[23] Huang X., Ke K., Jin W., Zhu Q., Mei R., Zhang R., Yu S., Shou L., Sun X., Feng J. (2022). Identification of Genes Related to 5-Fluorouracil Based Chemotherapy for Colorectal Cancer. Front. Immunol..

[24] Sullivan B.G., Lo A., Yu J., Gonda A., Dehkordi-Vakil F., Dayyani F., Senthil M. (2023). Circulating Tumor DNA is Unreliable to Detect Somatic Gene Alterations in Gastrointestinal Peritoneal Carcinomatosis. Ann. Surg. Oncol..

[25] Baumgartner J.M., Riviere P., Lanman R.B., Kelly K.J., Veerapong J., Lowy A.M., Kurzrock R. (2020). Prognostic Utility of Pre- and Postoperative Circulating Tumor DNA Liquid Biopsies in Patients with Peritoneal Metastases. Ann. Surg. Oncol..

[26] Leick K.M., Kazarian A.G., Rajput M., Tomanek-Chalkley A., Miller A., Shrader H.R., McCarthy A., Coleman K.L., Kasi P.M., Chan C.H.F. (2020). Peritoneal Cell-Free Tumor DNA as Biomarker for Peritoneal Surface Malignancies. Ann. Surg. Oncol..

[27] Baumgartner J.M., Raymond V.M., Lanman R.B., Tran L., Kelly K.J., Lowy A.M., Kurzrock R. (2018). Preoperative Circulating Tumor DNA in Patients with Peritoneal Carcinomatosis is an Independent Predictor of Progression-Free Survival. Ann. Surg. Oncol..

[28] Van’t Erve I., Rovers K.P., Constantinides A., Bolhuis K., Wassenaar E.C., Lurvink R.J., Huysentruyt C.J., Snaebjornsson P., Boerma D., van den Broek D. (2021). Detection of tumor-derived cell-free DNA from colorectal cancer peritoneal metastases in plasma and peritoneal fluid. J. Pathol. Clin. Res..

[29] Kastelein A.W., Vos L.M.C., van Baal J.O.A.M., Koning J.J., Hira V.V.V., Nieuwland R., van Driel W.J., Uz Z., van Gulik T.M., van Rheenen J. (2020). Poor perfusion of the microvasculature in peritoneal metastases of ovarian cancer. Clin. Exp. Metastasis.

